# Association between maternal and paternal employment and their children’s weight status and unhealthy behaviours: does it matter who the working parent is?

**DOI:** 10.1186/s12889-022-13735-3

**Published:** 2022-07-12

**Authors:** Néboa Zozaya, Juan Oliva-Moreno, Laura Vallejo-Torres

**Affiliations:** 1 Department of Quantitative Methods in Economics and Management, Universidad de Las Palmas de Gran Canaria, Calle Saulo Torón, 4 Las Palmas de Gran Canaria, 35017 Las Palmas, Spain; 2 Weber Economía Y Salud, Calle Moreto 17, 28014 Madrid, Spain; 3 Department of Economic Analysis and Finance, Universidad de Castilla La Mancha, Cobertizo de San Pedro Mártir, s/n, 45002 Toledo, Spain; 4 Department of Quantitative Methods in Economics and Management, Universidad de Las Palmas de Gran Canaria, Calle Saulo Torón, 4 Las Palmas de Gran Canaria, 35017 Las Palmas, Spain

**Keywords:** Childhood, Obesity, Unhealthy habits, Maternal employment, Parental employment, Multi-level

## Abstract

**Background:**

The growing number of employed women has been associated with an increase in the prevalence of overweight and obesity in children. We sought to determine whether childhood overweight/obesity in Spain is associated with labour participation of mothers and fathers, and whether the identity of the main caregiver has an influence on child’s weight and unhealthy behaviour.

**Methods:**

We used microdata from the 2010 and 2014 Health Behaviour in School-Aged Children surveys performed in Spain (*n* = 32,694). Logistic and linear multi-level regression models were applied to assess the association between parental employment and children’s self-reported weight status, accounting for school effects and controlling for socioeconomic factors. Separated binary models were also fitted for consumption of fruit, sweets, screen viewing and sedentarism.

**Results:**

In most cases, the significant associations between children’s weight and their parents’ work status disappeared once the models were adjusted for family wealth and education. However, we found persistent associations for some groups. Girls under 13 years-old living in households where the mother was the only employed parent were more likely to be affected by obesity and to report a higher body mass index value. Children in this type of household were more likely to show unhealthy lifestyles related to diet and leisure time activities.

**Conclusions:**

Parents’ socioeconomic characteristics had a protective effect on their children’s risk of obesity. Unhealthy behaviours were observed in households with a non-working father and a working mother, although the link with obesity was limited to girls. Our results suggest the need for a more equally shared burden of caregiving.

**Supplementary Information:**

The online version contains supplementary material available at 10.1186/s12889-022-13735-3.

## Background

An escalating global epidemic of overweight and obesity—“globesity”- is taking over many parts of the world [1], representing serious challenges for public health [2] due to its association with several chronic diseases [3]. Obesity during childhood is a major health concern, given its growing prevalence and long-term health consequences [4]. In 2016, over 340 million children and adolescents aged 5–19 were affected by overweight or obesity worldwide [5]. In Europe, although the prevalence may have stabilized [6], the differences between countries are especially striking, with a prevalence that ranges from more than 40% in southern Europe to less than 10% in northern Europe [7]. In Spain, despite the traditional Mediterranean diet, 28.6% and 10.3% of children between 2 and 17 years old are categorised as overweight and obese, respectively [8]. The prevalence of obesity among children shows a marked social gradient in Spain; 15.4% of children in the lowest social class are affected by obesity compared to 5.4% in the highest social class [9].

The direct cause of obesity is an energy imbalance between calories consumed and calories expended. This simple explanation is related to deep and complex social and technological changes which indirectly affect both diet and physical activity (at school, at work, at home and in leisure time) [10–14]. One of the social changes that is commonly referred to as a potential indirect cause of the increased overweight and obesity in children is the higher proportion of working women [15]. This implies major changes in family decisions about the division of time between paid work, domestic work and leisure, where “domestic work” includes both the time devoted to preparing meals and the time spent on the education and care of the children, among other tasks.

However, there are competing theoretical arguments about the direction of the relationship between parental employment and childhood overweight or obesity. Theoretically, parents who have a job would have less time to prepare meals with fresh food and supervise the quality and frequency of their children’s food intake. Skipping breakfast, eating fast food, and a quicker eating pace are all positively associated with childhood obesity [16]. Also, less parental supervision may discourage their children’s physical activity in early ages, leading to higher body mass index (BMI) [17]. However, having a job is also associated with higher income and educational levels, which are in turn linked to lower BMI.

In fact, the existing literature has found conflicting results in this area. There is certainly a body of studies that have identified a positive and significant relationship between maternal employment and the probability of children to be affected by overweight or obesity [17–24]. However, in other studies this result is tempered by the characteristics of the job and the number of hours worked, showing a lower effect or even a non-statistically significant relationship when these factors are controlled for [15, 25–29]. In addition, the increased involvement of fathers in raising children has been linked with a decreased likelihood that their children would become obese [30], which might offset any potential detrimental effect of the increase in maternal paid working hours. In fact, other studies have found that socioeconomic characteristics are more relevant determinants of children’s weight status [28, 31–37]. Moreover, the school environment has also been found to play a significant role in tackling obesity and promoting healthy lifestyles among children and adolescents [38–41]. Schools might create environments in which children eat healthfully and engage regularly in physical activity, leading to physical, emotional, and social benefits [39, 42]. They can also raise awareness and understanding of health risks (e.g. bullying and smoking), increase self-esteem and resistance to social pressure and promote healthy social relationships [43]. Disadvantaged and low academic performing schools are doubly burdened with additional risks, such as higher obesity risk [38]. There has been an emphasis on “whole school” interventions on health, which go beyond individual-focused education, and involve changes to schools’ overall organization, teaching, discipline, school health services, policies, culture, and extra-curricular activities [44, 45].

Our objective was to contribute to the understanding of the complex relationship between children’s excess weight and parental employment. We investigated whether overweight/obesity among children and adolescents in Spain is connected with parental employment, considering separately the situations in which a) only one parent (mother or father) has a job, b) both parents have a job, c) neither of the parents has a job. This approach allows us to explore more deeply the relationships between socioeconomic circumstances and parental employment, and to account for the roles of both fathers and mothers as carers of their children. Our analyses also aimed to determine the extent to which the school’s environment is associated with a higher weight. We also examined whether several unhealthy behaviours among children may be associated with parental employment status, taking into account the socioeconomic context of the family.

## Methods

### Sample

We used microdata from two consecutive waves of the Health Behaviour in School-Aged Children (HBSC) survey performed for Spain, for the years 2010 and 2014. Data were provided by the Spanish Ministry of Health, Consumption and Social Welfare [46]. HBSC is an international standardized cross-sectional survey supported by the WHO and aimed at understanding young people’s health-related behaviour, well-being, and developmental contexts [47]. In accordance with standard instructions and sampling procedures, responses were collected by means of standardised self-completed questionnaires administered in school classrooms, supported by teachers [48, 49]. The students were enrolled at a total of 532 representative educational centres in the 17 Spanish Autonomous Communities (plus two Autonomous Cities).

Our sample consisted of 32,694 students aged between 9 and 21 years belonging to biparental families (i.e., children who reported living with their father and mother; 79.93% of the whole sample), and was designed to allow the analysis of the working status of fathers and mothers separately.

### Variables

The outcome variable of our study was excessive weight among children/adolescents, and our calculations were based on the Body Mass Index computed from the weight and height data provided by the students. For adults, overweight is defined as having a BMI of 25 kg/m2 or higher (obesity as BMI ≥ 30 kg/m2) [50]. However, BMI in childhood changes substantially with age, and a wide variety of definitions of excess body fat in children are in use [51, 52]. For this study, we used the definitions of obesity and overweight specified by the WHO [53], using their cut-off points for BMI by sex and age, established at + 1 Standard Deviation (SD) for overweight and + 2SD for obesity. The consistency of the results was tested by using, as an alternative measure of obesity and overweight, the cut-off points for BMI proposed by Cole et al. (2000) [54]. We considered three dependent variables: obesity in a binary form (= 1 if obesity, and 0 otherwise), obesity together with overweight in a binary form (= 1 if overweight or obesity, and 0 otherwise), and BMI in a linear form. Outliers (BMI < 12 or BMI > 36) were excluded from the analysis (*n* = 295 or 1.01% of the initial database) [55].

Our explanatory variable of greatest interest was parental working status, which was based on responses directly reported by the children. We considered working parents as those whose children reported to “*have a job*”, while non-working parents might include different situations: he/she is sick, retired or a student; he/she is looking for a job; he/she cares for others or stays at home full time (househusband/housewife). Based on this information, and in order to explore the role of fathers’ and mothers’ working status separately, we defined parental employment taking into account four different situations: both parents had a job, only the father had a job, only the mother had a job, neither parent had a job at the time the survey. When only one of the parents had a job, we considered the non-employed parent as the most likely main caregiver of the child.

Several individual factors such as the child’s age, sex, number of siblings and parents’ country of birth were used as control variables, as well as dummy variables related to socioeconomic circumstances, the survey’s wave year and the region of residence of the child (Table 1). Socioeconomic factors included the educational level of the parents and the family’s material wealth. The latter was assessed using the Family Affluence Scale (FAS) [48]. An overall score was calculated as the sum of the following individual scores [56, 57]: car ownership (No: 0 points; Yes, one: 1 point; Yes, two or more: 2); having one’s own bedroom (No: 0 points; Yes: 1 point); number of computers/laptops at home (None: 0 points; One: 1 point; Two: 2 points; More than two: 3 points); and number of family holidays during the past year (None: 0 points; One: 1 point; Two: 2 points; More than two: 3 points). Using an additive score, the responses were divided into three groups, following previous studies [57]: low (0–2 points); medium (3–5 points), and high (6–9 points) family wealth.

**Table 1:** Variables included in the main and auxiliary analysis

Area	Variables	Coding of the variables
**Dependent variables (main analysis)**		
Obesity		1 if the child is affected by obesity, 0 otherwise
Obesity / overweight		1 if the child is affected by obesity or overweight, 0 otherwise
BMI		Continuous variable between 12 and 35 kg/m2
**Dependent variables (auxiliary analysis)**		
Low fruit consumption		1 if the child usually doesn’t eat fruit every day of the week, 0 otherwise
Consumtion of sweets		1 if the child usually eats sweets or chocolate every day, 0 otherwise
Screens viewing		1 if the child usually watches TV, uses the computer/tablet or plays with the console for 4 h or more per day, 0 otherwise
Sedentarism		1 if the child didn’t feel physically active at least twice per week in the last 7 days, 0 otherwise
**Independent variables**		
**Variables of interest**		
**Parents’ working status**		Both parents have a job (dual-earner households) Only the father has a job Only the mother has a job Reference category: Neither of the parents has a job
**Control variables**		
**Individual**	Sex	1 if boy, 0 if girl
	Age	Continuous variable between 9 and 21 years old
**Socioeconomic**	Parents’ educational level	Both parents have university education (completed or not) Both parents have a primary level of education or no studies Reference category: the remaining situations (both parents have secondary studies or each of them has a different educational level)
	Family material wealth (FAS score)	1 if the child belongs to a family with medium or low wealth (less than 6 points at the FAS scale), 0 otherwise
	Parents’ origin	1 if both parents were born in Spain, 0 otherwise
	Siblings	Number of siblings
**Region**		18 dummies for each Spanish region (17 Autonomous Communities and 1 for Ceuta and Melilla, the two Spanish Autonomous Cities)
**Variables used to anchor the multi-level analysis**		
**Contextual variable**	School attended by the child	Dummies for each school: 133 for 2010 and 399 for 2014

At the supplementary file we show how questions were phrased at the survey

Besides the main analysis, auxiliary models were used to explore the underlying mecanisms or obesogenic behaviours that might explain childhood obesity/overweight. We tested whether unhealthy food habits (low fruit consumption and consumption of sweets) and leisure-time activities (screens viewing and sedentarism) among children were affected by their parental working status (Table 1).

### Statistical analysis

Pearson's chi-square test was used to determine the association between BMI categories and socioeconomic components. Logistic multi-level regression models were applied to assess the association of parental employment with the child’s weight status and behaviours. We used two-level models with random intercepts where children were nested within schools, and therefore the residual variance could be partitioned into a between-school component that represented unobserved school characteristics that affect children obesity outcomes, and a within-school component, representing the variance of children-level residuals. Separate models were used for the two alternative binary outcome variables (obesity and obesity + overweight), and linear models were fitted for BMI. Separate binary logistic multi-level models were also fitted for each unhealthy behaviour: low fruit consumption; consumption of sweets; screens viewing; and sedentarism (see Table 1).

All models controlled for sex, age, parents’ origin, number of siblings, region of residence and year (Model 1). For each dependent variable, different models were fitted, adding the family’s material wealth (Model 2), the parents’ educational level (Model 3) or both (Model 4). Separate models by sex and age groups (< 13 years old; 13–15 years old; 16 years old and more) were also fitted, based on the ordinary age of transition from primary to secondary education and the age of compulsory education in Spain [58]. To maximise the sample size, we created two dummy variables that included missing values for family’s material wealth and parental education. Interaction terms between parents’ work status and education/wealth were explored, but they were found to be non-significant, and thus they were not included in the analysis presented in this paper.

To quantify the between-school variation in obesity/overweight in the multi-level logistic regressions, median odds ratios (MOR) were calculated, while residual intra-class correlation coefficients (ICC) were estimated for the linear regressions in BMI [59]. MOR show the extent to which the individual probability of having obesity/overweight is determined by the school that the child attends. The Wald chi2 test was used for each predictor to assess whether the differences were significant. *P*-values lower than 0.05 were considered statistically significant. The analyses were performed using the Stata 14.2 program.

## Results

### Descriptive analysis

4.3% of the boys and 2.1% of the girls in the sample were affected by obesity. Most (58.2%) of the biparental families were considered as having high material wealth under the FAS measure. However, less than 19% of the families had both parents with university education. Regarding the work status, in 63.6% of the families both parents had a job, compared to 26.5% in which only the father had a job and 5.9% in which only the mother had a job. Almost 85% of the families were formed by parents born in Spain (Table 2).

**Table 2:** Prevalence of the sample characteristics and presence of obesity or overweight among the children (biparental families, 2010–2014)

		Prevalence	% with obesity	Pearson Chi2 test	% with overweight	Pearson Chi2 test
Sex	Boys	49.87	4.29	*p* = 0.000	18.38	*p* = 0.000
	Girls	50.13	2.08		11.53	
Age groups	Under 13 years	29.96	4.68	*p* = 0.000	18.65	*p* = 0.000
	Between 13 and 15 years	46.78	2.67		14.04	
	16 years and older	23.78	2.34		12.22	
Economic level	Low family affluence	2.20	4.06	*p* = 0.000	15.23	*p* = 0.001
	Medium family affluence	39.60	3.98		16.10	
	High family affluence	58.21	2.65		14.16	
Parental working status	Both parents have a job	63.62	2.94	*p* = 0.000	14.77	*p* = 0.850
	Only the father has a job	26.53	3.21		15.03	
	Only the mother has a job	5.94	4.15		15.35	
	Neither of the parents has a job	3.91	4.97		15.37	
Parental educational level	Both of university level	18.66	2.10	*p* = 0.000	12.77	*p* = 0.000
	Both of secondary level or each parent of a different level	59.86	2.86		14.74	
	Both of primary level or less	21.48	4.12		16.22	
Parents’ origin	Both parents born in Spain	84.73	3.04	*p* = 0.013	14.59	*p* = 0.000
	One parent born in Spain	5.33	3.17		17.33	
	Neither of the parents born in Spain	9.94	4.08		16.71	
Siblings	Only child	15.22	3.12	*p* = 0.000	16.32	*p* = 0.007
	One brother/sister	60.28	2.93		14.93	
	Two brothers/sisters	16.44	3.56		14.48	
	Three or more brothers/sisters	8.06	4.47		13.27	

Overweight and obesity prevalences varied by age group, sex and socioeconomic characteristics. The child’s weight status was associated with his/her parents’ economic and educational level (*p* < 0.001). The prevalence of obesity was lowest among children who reported having two employed parents. Children with only a working father showed a lower prevalence of obesity than children with a working mother only (3.21% versus 4.15%) (Table 2).

There are large and statistical significant associations between the family’s wealth and the work status of parents. In wealthy biparental families, more than 69% of the households had two working parents, whereas this rate drop to 56% among medium- or low-wealth families. The proportion of wealthy families with a working mother and a non-working father almost halves that of less wealthy families (3.90% versus 7.86%). The educational background of parents is also closely linked to their work status (Table 3).

**Table 3:** Prevalence of families’ socioeconomic level, by parents’ work status (biparental families, 2010–2014)

	% Both parents have a job	% Only the father has a job	% Only the mother has a job	% Neither of the parents has a job	
High wealth (FAS 3) (*n* = 12,242)	69.47	24.81	3.90	1.82	100%
Medium or low wealth (FAS 1 or FAS_2) (*n* = 8,744)	55.63	30.10	7.86	6.42	100%
Pearson Chi2 test	*p* = 0.000				
Both parents with university education (*n* = 5,508)	78.25	17.50	3.32	0.93	100%
Both parents with secondary level education or each parent with a different level (*n* = 17,492)	63.10	27.46	6.13	3.32	100%
Both parents with primary education or less (*n* = 6,234)	50.22	32.72	8.39	8.66	100%
Pearson Chi2 test	*p* = 0.000				

In addition, children’s reported reasons why fathers do not have a job were different from the reasons provided for non-employed mothers. According to the children’s self-report, 67.98% of mothers in biparental families who do have a job are housewives or take care of others, while this proportion is 4.75% among non-working fathers.

### Regression analyses of children’s weight status

Sex and age are significant variables in all models (see Additional file 1). Being a boy is related to a higher risk of being affected by obesity or overweight than being a girl. Older children and children born to Spanish parents are less likely to be overweight or to have a higher BMI. A higher number of siblings is associated with a lower BMI and risk of overweight.

Living in households where neither of the parents has a job is associated with higher BMI values and a higher risk of being a child with obesity than living in dual-earner households (see Model 1 in Table 4). This result is retained when the model controls for family wealth (Model 2), but it disappears when we include additional controls for parental educational level (Model 4). In the simplest models (Model 1), we observe that children in households where only the mother has a job have a higher risk of obesity and higher BMI values than children whose both parents have a job. However, this result disappears after controlling for educational level and family wealth. Compared with living in a dual-earner household, households where the father is the only employed parent have not significantly different outcomes regarding children’s obesity. When using Cole’s alternative way of measuring obesity and overweight, the association of parental working status was not significant even in the simplest models (see Additional file 2).

**Table 4:** Multilevel regression models on children’s weight status

	Obesity				Obesity / overweight				BMI (linear)			
	Model 1	Model 2	Model 3	Model 4	Model 1	Model 2	Model 3	Model 4	Model 1	Model 2	Model 3	Model 4
Only the father has a job	1.029	1.005	0.977	0.962	1.022	1.009	0.991	0.983	0.013	-0.001	-0.027	-0.036
	(0.086)	(0.084)	(0.082)	(0.081)	(0.039)	(0.038)	(0.038)	(0.037)	(0.042)	(0.042)	(0.042)	(0.042)
Only the mother has a job	1.320**	1.259	1.231	1.188	1.093	1.065	1.051	1.031	0.218***	0.189**	0.168**	0.148
	(0.186)	(0.178)	(0.174)	(0.168)	(0.075)	(0.073)	(0.072)	(0.071)	(0.078)	(0.078)	(0.078)	(0.078)
None of the parents have a job	1.560***	1.438**	1.363	1.285	1.184**	1.134	1.096	1.063	0.258***	0.211**	0.164	0.131
	(0.249)	(0.230)	(0.220)	(0.208)	(0.100)	(0.096)	(0.093)	(0.091)	(0.098)	(0.098)	(0.098)	(0.099)
Medium–low family affluence		1.497***		1.418***		1.246***		1.205***	0.245*** (0.046)		0.201*** (0.046)	
		(0.130)		(0.124)		(0.050)		(0.049)				
Parents’ high educational level			0.657***	0.677***			0.785***	0.798***			-0.325***	-0.308***
			(0.076)	(0.079)			(0.038)	(0.039)			(0.051)	(0.051)
Parents’ low educational level			1.373***	1.334***			1.202***	1.183***			0.215***	0.197***
			(0.122)	(0.119)			(0.051)	(0.050)			(0.049)	(0.049)
Spanish parents	0.837	0.847	0.839	0.847	0.820***	0.826***	0.819***	0.824***	-0.247***	-0.239***	-0.247***	-0.240***
	(0.081)	(0.082)	(0.082)	(0.082)	(0.038)	(0.038)	(0.038)	(0.038)	(0.054)	(0.053)	(0.053)	(0.053)
MOR (school)	1.53	1.51	1.50	1.48	1.31	1.30	1.29	1.29	0.020	0.019	0.018	0.018
Wald Chi2 test	265	288.7	303.3	320.4	642.1	672.5	700.3	721.4	3285	3339	3428	3463

Odds ratios for obesity and obesity + overweight, and coefficients for BMI

Robust seeform in parentheses. *** *p* < 0.01, ** *p* < 0.05

All models were also adjusted for gender, age, number of siblings, region and year. Models 2 and 4 were also adjusted for the missing variables of the family’s socioeconomic level. Models 3 and 4 were also adjusted for the missing variables of the parent’s educational level. Only biparental families considered. *N* = 27,598. 532 schools

Reference categories: both parents have a job; high family affluence; parents’ medium educational level; non-Spanish parents

With regards to socioeconomic and environmental factors, we observe a significant association of family’s wealth/education and the school environment with children’s weight status. Children and adolescents in poorer households are more likely to have obesity and/or overweight in every model. Parents with higher education have a protective influence over their children’s weight status; the children of parents with university degrees are less likely to be affected by obesity/overweight and have lower BMI values, while the opposite is true for children whose parents have only primary education or less.

According to the MOR values obtained from the models, if children move to a school with a higher probability of obesity/overweight, their risk of having obesity and/or overweight will (in median) increase between 1.29 and 1.53 times. This impact is slightly greater than the household wealth effect estimated in the models.

Separate analyses by sex and age groups reveal similar patterns, with some exceptions. Girls under 13 years old show a significantly higher risk of being affected by obesity or having a greater BMI when their mothers are the only working parent, even after controlling for the socioeconomic and educational level of their family. The effect of belonging to this type of household on continuous BMI is also significant and quantitatively greater among older girls (aged between 13 and 15 years old), whereas the effect among boys is not significant. Boys aged between 13 and 15 years old with two working parents have a significant lower risk of obesity than those who reported living in households where neither of the parents has a job (Table 5).

**Table 5:** Multilevel regression models on children’s obesity/BMI, by sex and age group

	Boys under 13		Girls under 13		Boys 13–15 years old		Girls 13–15 years old		Boys ≥ 16 years old		Girls ≥ 16 years old	
	Obesity	BMI	Obesity	BMI	Obesity	BMI	Obesity	BMI	Obesity	BMI	Obesity	BMI
Only the father has a job	1.159	0.101	1.123	0.158	1.051	-0.124	0.731	0.070	0.667	-0.189	0.693	-0.217
	(0.180)	(0.112)	(0.272)	(0.108)	(0.169)	(0.093)	(0.169)	(0.084)	(0.177)	(0.121)	(0.234)	(0.126)
Only the mother has a job	1.270	0.105	3.504***	0.870***	1.067	-0.260	0.828	0.425***	1.049	0.116	0.429	0.061
	(0.386)	(0.233)	(1.144)	(0.210)	(0.297)	(0.170)	(0.338)	(0.159)	(0.438)	(0.216)	(0.268)	(0.206)
None of the parents have a job	1.638	0.424	0.743	0.019	1.972**	0.308	0.311	-0.048	1.617	0.024	1.182	0.342
	(0.559)	(0.302)	(0.476)	(0.290)	(0.564)	(0.225)	(0.195)	(0.194)	(0.670)	(0.256)	(0.549)	(0.245)
Medium–low family affluence	1.442**	0.299**	1.298	0.212	1.258	0.254**	1.545	0.243***	1.551	0.140	1.502	0.045
	(0.240)	(0.122)	(0.351)	(0.116)	(0.209)	(0.102)	(0.358)	(0.090)	(0.399)	(0.130)	(0.514)	(0.134)
Parents’ high educational level	0.712	-0.234	1.108	-0.240	0.544***	-0.455***	0.607	-0.371***	0.641	-0.356**	0.576	-0.474***
	(0.150)	(0.127)	(0.327)	(0.125)	(0.126)	(0.109)	(0.212)	(0.105)	(0.238)	(0.151)	(0.320)	(0.163)
Parents’ low educational level	1.474**	0.559***	1.552	0.349**	1.193	0.197	1.379	0.220**	1.064	0.047	1.631	0.233
	(0.287)	(0.149)	(0.443)	(0.144)	(0.199)	(0.106)	(0.307)	(0.094)	(0.273)	(0.134)	(0.477)	(0.129)

Odds ratios for obesity and coefficients for BMI. Robust seeform in parentheses. *** *p* < 0.01, ** *p* < 0.05

All models were adjusted for region, parents’ origin, number of siblings, year, family affluence, parents’ educational level, the missing variables of the family’s socioeconomic level and the missing variables of the parents’ educational level. Only biparental families were considered

Reference categories: both parents have a job; high family affluence; parents’ medium educational level

### Auxiliary models: regression analyses on unhealthy lifestyles

According to the auxiliary models used for the potential correlated factors of excess weight in children, sedentarism is found to be significantly higher in households other than those where both parents were employed, after controlling for household socioeconomic characteristics (Table 6). Children living in households where only the mother has a job are less likely to eat fruit daily, and are more likely to eat sweets and view screens daily than children who belong to families with other work characteristics, even after controlling for family wealth and education.

**Table 6:** Multilevel regression models on unhealthy lifestyles (Odds ratios)

	Low fruits consumption	Sweets consumption	Screens viewing	Sedentarism
Only the father has a job	1.038	1.052	1.029	1.143***
	(0.030)	(0.042)	(0.050)	(0.040)
Only the mother has a job	1.121**	1.165**	1.197**	1.247***
	(0.061)	(0.083)	(0.104)	(0.078)
None of the parents have a job	1.037	1.147	1.191	1.352***
	(0.071)	(0.098)	(0.125)	(0.101)
Medium–low family affluence	1.190***	0.986	0.985	1.161***
	(0.038)	(0.043)	(0.044)	(0.044)
Parents’ high educational level	0.631***	0.916	0.672***	0.872***
	(0.021)	(0.047)	(0.046)	(0.040)
Parents’ low educational level	1.202***	1.130***	1.179***	1.230***
	(0.041)	(0.050)	(0.063)	(0.047)
Spanish parents	1.047	0.842***	0.781***	0.834***
	(0.038)	(0.041)	(0.046)	(0.035)
Observations	30,427	30,240	18,727	29,840
Number of groups	532	532	526	532
Wald Chi2 test	677	253	215	1191
MOR (school)	1.26	1.33	1.40	1.20

seEform in parentheses. *** *p* < 0.01, ** *p* < 0.05

All models were adjusted for region, parents’ origin, number of siblings, year, family affluence, parents’ educational level, the missing variables of the family’s socioeconomic level and the missing variables of the parents’ educational level. Only biparental families were considered

Reference categories: both parents have a job; high family affluence; parents’ medium educational level

Frequent consumption of fruit and physical activity are linked to wealthy families, while a lower educational background of the parents seems to be a negative factor for all analysed correlated factors of their child’s obesity. Children of Spain’s native parents are also less likely to undertake unhealthy behaviours that can lead to obesity than children of non-Spanish parents. In these models, the residual heterogeneity between schools (MOR 1.20–1.40) is found to be of greater relevance than the impact of family wealth.

## Discussion

Our results indicate that family’s socioeconomic characteristics are determining factors of obesity and unhealthy habits among children. In most cases, these factors overweighted the detrimental effect that the potential lower parental supervision expected in households with working parents might have on children’s weight and obesogenic behaviours. However, in some cases, the labour participation of mothers was found to be linked to higher children’s obesity and unhealthy habits when mothers were the only providers of the households, suggesting that the availability of a non-working father did not offset the impact of maternal employment. To our knowledge, this is the first study that examines the underlying factors that affect children’s weight status, differentiating by parental work status and including the potential effect of the school as an additional level of analysis.

Our results confirm that, in line with previous studies, family’s educational level [31, 33, 37] and incomes are highly associated with childhood obesity/overweight [28, 36, 60], as well as the school environment [38–41]. In Spain, an immigrant background of the parents has also been associated with obesity among children [35].

The findings that maternal employment is not associated with children’s weight status or unhealthy behaviours when both parents are employed but, for at least some subgroups, working mothers are associated with a higher prevalence of child’s obesity and unhealthy habits when they are the only providers of the household is striking. This may reinforce the conclusions of other studies claiming that mothers, even when they are working outside the home, assume greater responsibility for child care and home management than fathers do [21, 61]. In addition, a closer look at the characteristics of biparental households with only a working mother indicates that these families are likely to be categorized as having limited financial resources, suggesting that unobserved socioeconomic factors might also play a role in the observed association.

The detrimental association of a non-working father on children’s weight and lifestyles, while observing that having a non-working mother is not associated with poorer outcomes also deserves further discussion. This finding might suggest that having a mother as main caregiver may have a greater positive influence on children’s weight and lifestyles than having a father as main caregiver. However, our results should be interpreted in the light of the socioeconomic context at the time the data was collected. Both in 2010 and in 2014 the difference in the level of unemployment between men and women was the smallest in the history of Spain. This was due to the more severe impact of the economic crisis on sectors that employ more men than women. As a result, the unemployment rate among men increased from 6.4% in 2007 to 25.6% in 2013 [62]. This suggests that non-working fathers in our data might in some cases represent newly unemployed men affected by the past recession. In fact, in our data children reported that 59% of non-working fathers were looking for a job, while this percentage was 29% among non-working mothers (however, around 10% of children did not know the reason why their father/mother did not have a job). The potential benefits of the expected increased involvement of fathers in their children’s raising might be fewer in such circumstances.

Our study has certain limitations that should be mentioned. First, given the nature of the data used, it is difficult to establish a causal relationship between parents’ work status and children’s BMI, as parental labour force is likely to be correlated with a number of factors that are also related to children’s weight (e.g. educational background, parent’s weight status, etc.). Second, our analyses are based on the information contained in the HBSC cross-sectional surveys, which albeit of being the richest source of data on children’s health in Spain, the information provided is limited (i.e. there was no information on the number of working hours) and self-reported. Therefore, our estimates might be affected by measurement errors and by omitted variables bias, making causal inferences problematic. Regarding measurement errors, children and adolescents completed the questionnaires on their own using an online platform at their school centres with support from school teachers. However, the self-reported nature of the data might lead to some discrepancies with reality regarding height, weight, family structure and parents’ work status and educational level, as well as regarding the elements needed to estimate family affluence, dietary behaviour, leisure-time habits, etc., especially among younger children. A previous study analysed the validity of self-reported height and weight data within the framework of the HBSC study conducted in Estonia and found a small and age-decreasing underestimation of overweight/obesity prevalences when compared with measured anthropometric data [63]. In Spain, obesity and overweight prevalences estimated by the Spanish National Health Survey in 2011/12 showed very similar values for the age groups included in the HBSC [64]. With respect to the role of omitted variables, the factors included as control variables in our models might only be a subset of all possible factors related to employment and child’s outcomes. For instance, parents’ own health and weight status might affect their ability to have a job and be correlated with their children’s obesity outcomes. Therefore, we might expect that some degree of endogeneity is still affecting our estimates.

Finally, the reasons why fathers do not have a job seem to be different from the reasons for non-working mothers. This, together with our finding indicating that employed mothers might be linked to higher children’s obesity and unhealthy habits when they are the only working parent in the household, emphasises A more balanced sharing of child’s care between men and women might weaken the documented link between maternal employment and children’s unhealthy behaviours and weight. Our results suggest the the need for a new social contract in which the burden of caregiving is equally shared, so that the participation of either parent in the employment market does not involve a risk for their children’s health. Conditions of employment must also favour the conciliation between family and professional life. According to the European Job Quality Index, which is “a measure that encompasses a broad range of work and employment characteristics, including wages, non-wage aspects of employment and work organisation, and collective interest representation” [65], Spain has the third worst position in this ranking. The socioeconomic and educational gradient which we observed in children’s obesity and unhealthy behaviours, as well as the effect of the school environment, also point towards the need to act at the root of the observed inequalities in children’s weight status. On this matter, the evidence shows that complex obesity prevention interventions acting on multiple targets, settings, and risk factors appear both to be more effective and to lower health inequalities than individual actions [66, 67].

## Conclusions

In conclusion, our findings indicate that once we control for family wealth and education, the observed association between a working mother and her children’s risk of obesity disappears, except for younger girls living in households where the mother had a job and the father did not. Unhealthy behaviours were also observed in households where the mother was the only employed parent. This study highlights the need for more complete research into the way in which obesity develops in young people, and into the roles that working and non-working parents have in their children’s development.

Further studies should be undertaken, including the use of time, to analyse the relationship between family members, and also to discern whether the results may change over time or working regimes. It would also be desirable to explore a larger number of potential causes of obesity, to assess the importance of reverse causality between education, income, and obesity, and to determine the nature of childcare among biparental families. Further research is also needed to widen the analysis to include families other than biparental ones, although the formers are more heterogeneous, and so more information about additional caregiving would be needed. As we get collectively closer to understanding the development of children’s excess weight, we may be better able to craft appropriate public health interventions to help reduce its current prevalence.

## Supplementary Information

**Additional file 1: Table S1.** Multilevel regression models on children obesity (Full models) Odds ratios. **Table S2.** Multilevel regression models on children obesity/overweight (Full models) Odds ratios. **Table S3.** Multilevel regression models on children BMI (Full models) Coefficients. **Table S4.** Multilevel regression models on children weight status measured by Cole et al (Odds ratios). HBSC questionnaire.

## Data Availability

Microdata from the HBSC surveys were provided by the Spanish Ministry of Health, Consumer Affairs and Social Welfare as well as by the University of Seville. Microdata is now available at the HBSC Data Management Centre: https://www.uib.no/en/hbscdata.

## Abbreviations

BMI: Body Mass Index
FAS: Family Affluence Scale
HBSC: Health Behaviour in School-Aged Children
ICC: Intra-class correlation coefficient
MOR: Median odds ratio
SD: Standard Deviation
WHO: World Health Organization
