# Supplementary material for: Association between maternal and paternal employment and their children’s weight status and unhealthy behaviours: does it matter who the working parent is?
Source: BMC Public Health. 2022 Jul 12;22:1331. doi: 10.1186/s12889-022-13735-3 (PMC9277834; doi:10.1186/s12889-022-13735-3)
Supplement: Supplementary file 1 — Additional file 1: Table S1. Multilevel regression models on children obesity (Full models) Odds ratios. Table S2. Multilevel regression models on children obesity/overweight (Full models) Odds ratios. Table S3. Multilevel regression models on children BMI (Full models) Coefficients. Table S4. Multilevel regression models on children weight status measured by Cole et al (Odds ratios). HBSC questionnaire. [file 12889_2022_13735_MOESM1_ESM.pdf]

Article title: Association between maternal and paternal employment and their children's weight status and unhealthy behaviours: does it matter who the working parent is?
Journal name: BMC Public Health
Authors' information: Néboa Zozaya[1], Juan Oliva-Moreno[2] and Laura Vallejo-Torres[3]

1. Universidad de Las Palmas de Gran Canaria, Department of Quantitative Methods in Economics and Management, Calle Saulo Torón, 4 Las Palmas de Gran Canaria, 35017. Las Palmas, Spain. Weber Economía y Salud, Calle Moreto 17, 28014 Madrid, Spain. Corresponding author: neboa.zozaya@weber.org.es
2. Universidad de Castilla La Mancha, Department of Economic Analysis and Finance, Cobertizo de San Pedro Mártir, s/n, 45002 Toledo, Spain.
3. Universidad de Las Palmas de Gran Canaria, Department of Quantitative Methods in Economics and Management, Calle Saulo Torón, 4 Las Palmas de Gran Canaria, 35017 Las Palmas, Spain.

Additional file 1. Tables

Table S1. *Multilevel regression models on children obesity* (Full models) Odds ratios

	Model 1	Model 2	Model 3	Model 4
Sex (boy)	2.189***	2.203***	2.213***	2.224***
	(0.164)	(0.165)	(0.166)	(0.167)
Age	0.859***	0.856***	0.858***	0.856***
	(0.017)	(0.016)	(0.018)	(0.018)
Only the father has a job	1.029	1.005	0.977	0.962
	(0.086)	(0.084)	(0.082)	(0.081)
Only the mother has a job	1.320**	1.259	1.231	1.188
	(0.186)	(0.178)	(0.174)	(0.168)
None of the parents have a job	1.560***	1.438**	1.363	1.285
	(0.249)	(0.230)	(0.220)	(0.208)
Spanish parents	0.837	0.847	0.839	0.847
	(0.081)	(0.082)	(0.082)	(0.082)
Number of siblings	1.061	1.058*	1.053	1.052
	(0.036)	(0.036)	(0.036)	(0.036)
Medium-low family affluence		1.497***		1.418***
		(0.130)		(0.124)
Missing_family affluence		1.197*		1.173
		(0.119)		(0.117)
Year 2014	0.846	0.820*	0.904	0.879
	(0.088)	(0.090)	(0.099)	(0.101)
Region 2	0.660**	0.675**	0.690**	0.702
	(0.126)	(0.127)	(0.130)	(0.131)
Region 3	0.769	0.771	0.824	0.823
	(0.200)	(0.199)	(0.212)	(0.211)
Region 3	0.850	0.852	0.856	0.859
	(0.183)	(0.182)	(0.182)	(0.181)
Region 5	1.661**	1.662**	1.720***	1.721***
	(0.353)	(0.348)	(0.360)	(0.357)
Region 6	0.449***	0.451***	0.466***	0.467***
	(0.114)	(0.113)	(0.117)	(0.116)
Region 7	0.340***	0.342***	0.352***	0.353***
	(0.091)	(0.090)	(0.093)	(0.093)
Region 8	0.775	0.774	0.772	0.772
	(0.155)	(0.153)	(0.153)	(0.152)
Region 9	0.654	0.676	0.673	0.691
	(0.152)	(0.155)	(0.154)	(0.157)
Region 10	0.759	0.772	0.784	0.795
	(0.165)	(0.166)	(0.168)	(0.169)
Region 11	0.721	0.734	0.738	0.748
	(0.150)	(0.151)	(0.152)	(0.153)
Region 12	0.618**	0.621**	0.645**	0.646**
	(0.134)	(0.133)	(0.138)	(0.137)

Region 13	0.539**	0.553**	0.578**	0.588**
	(0.136)	(0.138)	(0.144)	(0.145)
Region 14	1.091	1.082	1.074	1.069
	(0.228)	(0.223)	(0.221)	(0.218)
Region 15	0.538**	0.543**	0.557**	0.562**
	(0.131)	(0.130)	(0.134)	(0.134)
Region 16	0.454***	0.457***	0.495***	0.495***
	(0.119)	(0.119)	(0.129)	(0.128)
Region 17	0.445***	0.451***	0.452***	0.457***
	(0.130)	(0.130)	(0.130)	(0.131)
Region 18	2.086***	2.225***	2.267***	2.380***
			0.657***	0.677***
Parents' high educational level			(0.076)	(0.079)
			1.373***	1.334***
Parents' low educational level			(0.122)	(0.119)
			1.189	1.192
Missing educational level			(0.168)	(0.168)
	0.251***	0.223***	0.235***	0.210***
Constant	(0.080)	(0.071)	(0.084)	(0.075)

Robust seeform in parentheses. *** p<0.01, ** p<0.05

Table S2. *Multilevel regression models on children obesity/overweight* (Full models) Odds ratios

	Model 1	Model 2	Model 3	Model 4
Sex (boy)	1.931***	1.938***	1.946***	1.950***
	(0.063)	(0.064)	(0.064)	(0.064)
Age	0.891***	0.890***	0.885***	0.885***
	(0.008)	(0.008)	(0.008)	(0.008)
Only the father has a job	1.022	1.009	0.991	0.983
	(0.039)	(0.038)	(0.038)	(0.037)
Only the mother has a job	1.093	1.065	1.051	1.031
	(0.075)	(0.073)	(0.072)	(0.071)
None of the parents have a job	1.184**	1.134	1.096	1.063
	(0.100)	(0.096)	(0.093)	(0.091)
Spanish parents	0.820***	0.826***	0.819***	0.824***
	(0.038)	(0.038)	(0.038)	(0.038)
Number of siblings	0.957**	0.955**	0.953***	0.952***
	(0.017)	(0.017)	(0.017)	(0.017)
Medium-low family affluence		1.246***		1.205***
		(0.050)		(0.049)
Missing_family affluence		1.124***		1.109**
		(0.050)		(0.049)
Year 2014	0.928	0.904*	0.940	0.917
	(0.049)	(0.050)	(0.051)	(0.052)
Region 2	0.673***	0.680***	0.680***	0.686***
	(0.067)	(0.067)	(0.066)	(0.067)
Region 3	0.755**	0.754**	0.773	0.771**
	(0.101)	(0.100)	(0.102)	(0.101)
Region 3	0.907	0.907	0.899	0.899
	(0.103)	(0.103)	(0.101)	(0.100)
Region 5	1.206	1.204	1.217	1.215
	(0.147)	(0.146)	(0.146)	(0.145)
Region 6	0.703***	0.706***	0.712***	0.713***
	(0.083)	(0.083)	(0.083)	(0.082)
Region 7	0.616***	0.618***	0.619***	0.620***
	(0.072)	(0.072)	(0.071)	(0.071)
Region 8	0.846	0.845	0.833	0.833
	(0.089)	(0.088)	(0.086)	(0.085)
Region 9	0.774**	0.784**	0.776**	0.784**
	(0.091)	(0.092)	(0.090)	(0.090)
Region 10	0.748**	0.753**	0.751**	0.755**
	(0.085)	(0.085)	(0.084)	(0.084)
Region 11	0.778**	0.784**	0.778**	0.783**
	(0.083)	(0.083)	(0.082)	(0.082)
Region 12	0.884	0.887	0.890	0.892
	(0.093)	(0.093)	(0.092)	(0.092)
Region 13	0.710***	0.718***	0.734**	0.739**
	(0.088)	(0.089)	(0.090)	(0.090)
Region 14	1.075	1.073	1.047	1.047
	(0.122)	(0.121)	(0.116)	(0.116)
Region 15	0.654***	0.655***	0.658***	0.660***
	(0.079)	(0.078)	(0.078)	(0.078)
Region 16	0.655***	0.655***	0.672***	0.672***
	(0.080)	(0.079)	(0.081)	(0.080)
Region 17	0.641***	0.645***	0.641***	0.644***
	(0.088)	(0.088)	(0.086)	(0.086)
Region 18	1.336	1.384**	1.390**	1.429**
	(0.221)	(0.227)	(0.226)	(0.232)
Parents' high educational level			0.785***	0.798***
			(0.038)	(0.039)
Parents' low educational level			1.202***	1.183***
			(0.051)	(0.050)
Missing educational level			0.998	0.999
			(0.072)	(0.072)
Constant	1.253	1.172	1.368	1.284
	(0.194)	(0.181)	(0.232)	(0.218)

Robust seeform in parentheses. *** p<0.01, ** p<0.05.

Table S3. *Multilevel regression models on children BMI* (Full models) Coefficients

	Model 1	Model 2	Model 3	Model 4
Sex (boy)	0.477***	0.480***	0.486***	0.487***
	(0.036)	(0.036)	(0.036)	(0.036)
Age	0.549***	0.548***	0.541***	0.540***
	(0.010)	(0.010)	(0.011)	(0.011)
Only the father has a job	0.013	-0.001	-0.027	-0.036
	(0.042)	(0.042)	(0.042)	(0.042)
Only the mother has a job	0.218***	0.189**	0.168**	0.148
	(0.078)	(0.078)	(0.078)	(0.078)
None of the parents have a job	0.258***	0.211**	0.164	0.131
	(0.098)	(0.098)	(0.098)	(0.099)
Spanish parents	-0.247***	-0.239***	-0.247***	-0.240***
	(0.054)	(0.053)	(0.053)	(0.053)
Number of siblings	-0.040**	-0.042**	-0.042**	-0.043**
	(0.020)	(0.020)	(0.020)	(0.020)
Medium-low family affluence		0.245***		0.201***
		(0.046)		(0.046)
Missing_family affluence		0.114**		0.094
		(0.049)		(0.049)
Year 2014	-0.206***	-0.229***	-0.191***	-0.211***
	(0.068)	(0.070)	(0.069)	(0.071)
Region 2	-0.510***	-0.497***	-0.497***	-0.488***
	(0.126)	(0.125)	(0.123)	(0.123)
Region 3	-0.345**	-0.344**	-0.319	-0.320
	(0.169)	(0.168)	(0.166)	(0.165)
Region 3	-0.287	-0.285	-0.301**	-0.298**
	(0.149)	(0.148)	(0.146)	(0.145)
Region 5	0.135	0.132	0.142	0.139
	(0.163)	(0.161)	(0.159)	(0.158)
Region 6	-0.420***	-0.416***	-0.408***	-0.405***
	(0.151)	(0.149)	(0.147)	(0.146)
Region 7	-0.650***	-0.646***	-0.649***	-0.646***
	(0.146)	(0.145)	(0.143)	(0.142)
Region 8	-0.277**	-0.277**	-0.300**	-0.298**
	(0.136)	(0.135)	(0.133)	(0.133)
Region 9	-0.438***	-0.420***	-0.437***	-0.423***
	(0.151)	(0.150)	(0.147)	(0.147)
Region 10	-0.232	-0.222	-0.227	-0.219
	(0.145)	(0.144)	(0.142)	(0.141)
Region 11	-0.420***	-0.410***	-0.422***	-0.413***
	(0.137)	(0.136)	(0.134)	(0.134)
Region 12	-0.158	-0.155	-0.154	-0.151
	(0.136)	(0.135)	(0.133)	(0.133)
Region 13	-0.601***	-0.587***	-0.560***	-0.550***
	(0.159)	(0.158)	(0.155)	(0.154)
Region 14	0.028	0.025	-0.009	-0.009
	(0.150)	(0.149)	(0.147)	(0.146)
Region 15	-0.443***	-0.439***	-0.432***	-0.429***
	(0.153)	(0.151)	(0.149)	(0.148)
Region 16	-0.361**	-0.356**	-0.329**	-0.327**
	(0.152)	(0.150)	(0.148)	(0.148)
Region 17	-0.801***	-0.792***	-0.802***	-0.795***
	(0.172)	(0.171)	(0.168)	(0.167)
Region 18	0.512**	0.551**	0.560***	0.590***
	(0.222)	(0.220)	(0.217)	(0.216)
Parents' high educational level			-0.325***	-0.308***
			(0.051)	(0.051)
Parents' low educational level			0.215***	0.197***
			(0.049)	(0.049)
Missing educational level			-0.024	-0.023
			(0.087)	(0.087)
Constant	12.731***	12.658***	12.861***	12.793***
	(0.186)	(0.186)	(0.200)	(0.201)

Robust seeform in parentheses. *** p<0.01, ** p<0.05

Table S4. *Multilevel regression models on children weight status measured by Cole et al* (Odds ratios)

	Obesity				Obesity + overweight			
	Model 1	Model 2	Model 3	Model 4	Model 1	Model 2	Model 3	Model 4
Only the father has a job	1.054<br>(0.100)	1.036<br>(0.099)	0.996<br>(0.095)	0.986<br>(0.095)	1.011<br>(0.038)	0.999<br>(0.038)	0.978<br>(0.037)	0.971<br>(0.037)
Only the mother has a job	1.111<br>(0.191)	1.071<br>(0.184)	1.031<br>(0.178)	1.005<br>(0.173)	1.090<br>(0.074)	1.063<br>(0.073)	1.046<br>(0.071)	1.027<br>(0.070)
None of the parents have a job	1.365<br>(0.258)	1.281<br>(0.244)	1.195<br>(0.229)	1.145<br>(0.220)	1.165<br>(0.097)	1.118<br>(0.094)	1.075<br>(0.091)	1.045<br>(0.088)
Medium-low family affluence		1.354***<br>(0.137)		1.278**<br>(0.130)		1.234***<br>(0.050)		1.190***<br>(0.048)
Parents' high educational level			0.586***<br>(0.082)	0.599***<br>(0.084)			0.757***<br>(0.037)	0.768***<br>(0.038)
Parents' low educational level			1.277**<br>(0.131)	1.250**<br>(0.128)			1.194***<br>(0.050)	1.176***<br>(0.050)
Spanish parents	0.796**<br>(0.088)	0.804**<br>(0.089)	0.798**<br>(0.089)	0.805<br>(0.089)	0.828***<br>(0.038)	0.833***<br>(0.038)	0.827***<br>(0.038)	0.832***<br>(0.038)
MOR (school)	1.51	1.49	1.47	1.46	1.31	1.30	1.29	1.28
Wald Chi2 test	142.2	152.6	171.2	177.7	412.8	440.8	477.8	496.5

Robust seeform in parentheses. *** p<0.01, ** p<0.05.
All models were adjusted for gender, age, region, number of siblings and year. Models 2 and 4 were also adjusted for the missing variables of the family's socioeconomic level. Models 3 and 4 were also adjusted for the missing variables of the parent's educational level. Only biparental families were considered.

Additonal file 2. HBSC questionnaire

Family structure:

All families are different (for example, not everyone lives with both their parents, sometimes people live with just one parent, or they have two homes or live with two families) and we would like to know about yours. Please answer this first question for the home where you live all or most of the time and tick the people who live there.

Adults	Children
○ Mother	Please say how many brothers and sisters live here (including half, step or foster brothers and sisters). Please write in the number or write 0 (zero) if there are none. Please do not count yourself
○ Father	
○ Stepmother (or father's girlfriend)	
○ Stepfather (or mother's boyfriend)	
○ Grandmother	How many brothers? ______
○ Grandfather	How many sisters? ______
○ I live in a foster home or children's home	
○ Someone or somewhere else: please write it down ______	

Body mass:

How much do you weigh without clothes? ______
How tall are you without shoes? ______

Parents' employment:

Father	Mother
**Does your father have a job?**	**Does your mother have a job?**
○ Yes	○ Yes
○ No	○ No
○ Don't know	○ Don't know
○ Don't know or don't see father	○ Don't know or don't see mother
**If YES, please say in what place he works** *(for example: hospital, bank, restaurant)* ______	**If YES, please say in what place she works** *(for example: hospital, bank, restaurant)* ______
**Please write down exactly what job he does there** *(for example: teacher, bus driver)* ______	**Please write down exactly what job she does there** *(for example: teacher, bus driver)* ______
**If NO, why does your father not have a job?** *(Please tick the box that best describes the situation)*	**If NO, why does your mother not have a job?** *(Please tick the box that best describes the situation)*
○ He is sick, or retired, or a student	○ She is sick, or retired, or a student
○ He is looking for a job	○ She is looking for a job
○ He takes care of others, or is full-time at home	○ She takes care of others, or is full-time at home
○ I don't know	○ don't know

Family's wealth:

Does your family own a car, van or truck?

○	No
○	Yes, one
○	Yes, two or more

Do you have your own bedroom for yourself?

○	No
○	Yes

During the past 12 months, how many times did you travel away on holiday *[vacation]* with your family?

○	Not at all
○	Once
○	Twice
○	More than twice

How many computers does your family own?

○	None
○	One
○	Two
○	More than two

Eating habits:

How many times a week do you usually eat or drink...?

	Never	Less than once a week	Once a week	2-4 days a week	5-6 days a week	Once a day, every day	Every day, more than once
Fruits	○	○	○	○	○	○	○
Vegetables	○	○	○	○	○	○	○
Sweets (candy or chocolate)	○	○	○	○	○	○	○

Physical activity:

Physical activity is any activity that increases your heart rate and makes you get out of breath some of the time.
Physical activity can be done in sports, school activities, playing with friends, or walking to school. Some examples of physical activity are running, brisk walking, rollerblading, biking, dancing, skateboarding, swimming, soccer, basketball, football, & surfing.
[COUNTRY SPECIFIC EXAMPLES CAN BE GIVEN]
For this next question, add up all the time you spent in physical activity each day.

Over the past 7 days, on how many days were you physically active for a total of at least 60 minutes per day?

0 days	1 day	2 days	3 days	4 days	5 days	6 days	7 days
○	○	○	○	○	○	○	○

Sedentary behaviour:

About how many hours a day do you usually use a computer for chatting on-line, internet, emailing, homework etc. in your free time? *Please tick one box for weekdays and one box for weekend.*

Weekdays		Weekend	
○	None at all	○	None at all
○	About half an hour a day	○	About half an hour a day
○	About 1 hour a day	○	About 1 hour a day
○	About 2 hours a day	○	About 2 hours a day
○	About 3 hours a day	○	About 3 hours a day
○	About 4 hours a day	○	About 4 hours a day
○	About 5 hours a day	○	About 5 hours a day
○	About 6 hours a day	○	About 6 hours a day
○	About 7 or more hours a day	○	About 7 or more hours a day
